# The geographical distribution of cancer.

**DOI:** 10.1038/bjc.1969.1

**Published:** 1969-03

**Authors:** R. Doll


					
BRITISH JOURNAL OF CANCER

VOL. XXIII              MARCII, 1969              NO. 1

THE GEOGRAPHICAL DISTRIBUTION OF CANCER

R. DOLL

From the Medical Research Council's Statistical Research Unit, University College

Hospital Medical School, 115 Gower Street, London, W.C.1

Received for publication October 7, 1968.

STUDY of the geographical distribution of cancer has long been recognized to
be one way of obtaining clues to its causation, and a great deal of work has been
carried out to determine the incidence of different types of cancer in different parts
of the world. Detailed incidence rates have been obtained for more than fifty
populations, either by national or regional cancer registries or by special investi-
gations in selected areas. Many of the results have been published in book form
or government reports which are not readily accessible, and others have been
published only in summary or in such a way that comparison with other sets of
data is difficult or impossible. To help overcome this, incidence data for thirty-
four populations in twenty-three countries have recently been brought together in
comparable form in a single volume by the International Union against Cancer
(1966).

Incidence data, however, constitute only part of the available evidence.
Mortality statistics have been published for the principal types of cancer in forty-
eight countries and in the urban areas of the fifteen Soviet republics (World
Health Organization, 1966; Segi and Kurihara, 1964; Merkova, Tserkovnogo, and
Kaufman, 1963) and, in some circumstances, these can provide useful indicators
of incidence. Indeed, in some areas in which cancer registration is incomplete,
mortality can provide a better indication of incidence than the official " incidence
statistics ", particularly for cancers whose fatality is high or for which it is possible
to obtain a reasonably accurate indication of the proportion that are not directly
responsible for causing death.

Finally, there is an immense number of clinical and pathological records.
Few can be used to provide reliable estimates of incidence, but, in exceptional
circumstances, they provide a clear indication of a situation that is qualitatively
different in different parts of the world (as with Burkitt's lymphoma and Kaposi's
sarcoma).

The extent of the variation in cancer incidence has been reviewed and the
geographical distribution of six types of cancer has been published in map form
elsewhere (Doll, 1967 and 1968). Corresponding numerical data and incidence
rates for ten other types of cancer are given here.

1

2                     R. DOLL

X a*s

~ A

* 0e

E  to

00

-ZZ-  o-

X co  o  0 0 .

t A-o i_  _    -

A e    ti

X   ? X 3 iM  s 0

GEOGRAPHICAL DISTRIBUTION OF CANCER

To allow for differences in the age distribution of different populations the
tabulated rates have been standardized for age, using a rounded off variant of
Segi's " world population " (Segi, 1960; International Union against Cancer,
1966). Standardization does not, however, overcome the difficulty that cancer
incidence varies with age in different ways in different countries nor, in particular,
for the fact that in some populations the incidence of many cancers levels off or even
falls in old age, whereas in other countries it continues to rise. This difference
can be due to cohort effects produced by changes in the prevalence of carcinogenic
factors with time or to other factors, such as the failure of old persons to make use
of the medical services. Comparisons have, therefore, been limited to the age
range 35 to 64 years, in which most cancers are relatively frequent, but which
excludes data for the oldest ages that are least likely to reflect current conditions
and are least likely to be accurate (Doll and Cook, 1967).

To enable mortality rates to be used, conversion factors have been calculated
from the data for eight countries that have published incidence and mortality rates
over approximately the same periods (Chile, Denmark, Finland, Israel, New
Zealand, Norway, Puerto Rico, and Sweden). These factors have been obtained
by dividing the relevant truncated incidence rates* (standardized for age) by the
corresponding mortality rates and averaging the result over the eight populations.
Estimates of cancer incidence in other countries have then been obtained by
multiplying the site-specific mortality rates by the corresponding factors. Judged
by the variability of the factors in the eight countries the error in the derived
incidence rates is, for the most part, likely to be less than 20 per cent (47 out of
70 observations) but it could rise to 30 per cent (8 out of 70 observations), and
the most extreme variations would be from -40 per cent to +68 per cent. For
some countries mortality rates have been published only for ten-year age groups
from 30 to 39 years of age rather than from 35 to 44 years of age. Other factors
have, therefore, been derived from British experience, using the pooled mortality
data for England and Wales over the ten years 1950 to 1959, to convert standard-
ized mortality rates at ages 30 to 59 years into the corresponding rates at ages
35 to 64 years. Both sets of factors are shown in Table I.

Table II gives incidence rates for fifteen types of primary cancer (13 in men and
2 in women) for thirty-four populations and for some of these cancers for a further
forty-three populations. For thirty-three populations the standardized incidence
rates have been derived from the age-specific rates given in the review volume
published by the International Union against Cancer (1966) and relate to periods
around 1960-62. Exceptionally the data for Denmark relate to 1953-57, for
Johannesburg to 1953-55, for Kyadondo County, Uganda, to 1954-60, and for
Singapore Chinese to 1950-61. Other sets of rates have been added for Bombay
(Bombay Cancer Registry, 1966) and for Indian and African populations in
Durban (Schonland and Bradshaw, 1968). For forty-one populations the rates
have been derived from the age-specific mortality statistics brought together by
the World Health Organization (1966), Segi and Kurihara (1964) and Merkova,
Tserkovnogo, and Kaufman (1963). These have been made comparable with the
incidence rates by multiplying by conversion factors (Table I) and, in sixteen
instances, by further conversion factors (Table I) to allow for the fact that the
published rates related to ages 30 to 59 years. These also have been chosen to
relate to periods close to or around 1961.

* That is, the rates for a selected age range, in this case usually 35 to 64 years.

3

R. DOLL

L.  0   O C O   O t
- I 0   C 0   0'   e q C

Iz   5 5   5 5i
COi

I 0 O  00  C-M C 0

CO0  C-0  OC   0

j>.. cot- eq 0 ? CO C- 00
COOl ? ? - ? ? CO CO

CO C- ? t-I - C'- CO CO 00
- - - ? eq ?-a - ?1 -

CO ? 00 CO 00 ? eq ?

- C- 00 CO CO CO 000
- - - - - - -

00 C- ? 0 0000 C- CO

- CO CO C- 00000 eq -
- - - eq - - -

eq00COoo00
?-400CO000C'.eq-
CO 00 C- ? ? 0 ? - -

- eq -

? eq 00 eq 0000

000 eq , 00CO eq eq CO

-4 CO 00 eq ? C.. CO CO

CO CO 00 ? 00 CO 0000

0000 C- C?- 00 CO CO C- 0

CO CO CO ? CO eq eq ?q eq

co to c =  "  -4  ,-aCt-Cto N C

0 00t40000"   COmCOcoI

I 1 I 1   I   I I 1I

0 0 0 0o              10 0I0 0 0

00CI O P0GP  "00  00 COf '4  4 -

o C 0004  MC O 001M 00CI1, -'

COCO C-OCOCO-

00 Ct- 0 eq -Id4 CO

00 ' C- C- C'O C-

t 0w 0w C-C-CO00C    00   q

C    OiLo ~ 0 4 I"   S  I  I CI 00I  I

0 0 C O O I C O C C - 0 C O 0 0   C 0 0  t . - e q ~

COCOOCOOCO0)OCOCO  00eq4 I-

eq CO6 CO CO 00 CO) Co t. CO

COi 004   0000    r .  e
eq eq eq eq r-

eq N e C OZ  Lo O  0000   .4

0 CO CO CO C- 11

CO Co r- C- CO C'-

eqi eq CO eq eq

CO 1" 00 Co Co CO

CO --  r-0  CO  Co

C0  - 00 1* 00 111

00 CO 00 CO CO 00

0~  CO  00~ eq~ 4   Ca

km eq CO q eq r-

I    I   I 1I I 1I I 1
I   I 1I 1 1I 1 1 I 1

I   I  I II   II  I

00 CO 00 CO C- eo C- eq

" eq 0 eq to COO CO.1

"   c   1  C O C O00N
"0co== I"00CxO

C O   C-   C O            0 0

I   IC O C O C -,   I I   II   I   c   I   I l

1-                        CO

r'- eq co

I  1  I    ,A~ I

0 00 000000 CO

" Co CO CO " 00

00 CO ll CO CO 00
CO C- LO 000 eqo

COr-  Cot  CO  eq  1

i   i1 i 1 1

11111111

0CLO0000CO00

Co 00 * I" Co eq CO -4

T- r- r- T- 7-

eq-4 Om  P00ONCO  C o 00  i00to 00  O co  q C-C co O000 CO OCOCOCO
eqeqC O C O~ 0 00C O C O   0 C 00 C O C O D  C -4 "   0   "   e q   00 0 -   " CO eq   0   00

-pG 6 1   o   oc   f   a L'   m   '   m . eqC  eq eq   qe   .q q   eq .   .   . C.O.  .

0-1 *0OW q0 eC-

"~CO 00 ~ 00e CO~ 00 CO

,   e q   C O   C O   C O   eq   0 0C - C

eq

1 1I 0 0 1 1 I 1  ~ ~

0   -4  cq   1-4 CO

II III  II  I

I  I  I1  I -I   il   I   I I   I   I l

.. .   .   . .... . .  . .. .-.. . ... . . . .

L a)   0*   a )   -

- )      .a). . .   . . .

cd   010 CL)  o  M  ce   ce~~~~~~~~~~~~~~~~~~~.0

4

C)
0

0-Q,
ZS

4Q,

p a b  4

ce q
E-a

.0.0
C0-4
0+.

0 0+

a)'
0eq
E CO

0,-

00

o   4C

GEOGRAPHICAL DISTRIBUTION OF CANCER

.*Ott-Wo0      O0OCOCOCOCO
COOCOCOOCD CODOeDoo-OCOaLCooCOc^Os

111111I I1    c00  OCOt-

Cot- Lo toC

I       I      I     I   I       I      I           I  I

-   -4  ?-I u:4 "4 O-   ?- O- 001   r-   r- r- r 0O x g X

0101010  00OE-0-_0c1DteXO01Ce>_Ot-

CO   0N   M-4 00 t-  CO CO   CO  CO
tC-t<-- 01VCoooo LOc t- mionOo co c _ls 0 co

COOMOCO 00 0 00 0 c OOeo  O L00 co  0

ClOOCIO  C XCC0 X*NOCOCO01CXNt1^>IOt4O

0  00  O Lo Ct  t-  O0  LO L  CO
|  |  |  | CA)  1G eq co  00 ||N || t-  r- 1:O  |  C

II  Ic I oo  OOOa4cQ  1  I  I  jctC =6o I I I 6C0o I t

COO  CO  0~~~01CoCo  c.  ~~~-~~0Co-4  r-it. C

Lo Co 01 co Co

t-: 41 CO CoO

C0 O C- N1 t-

_* C1 -H

010 CO c - C'-

LO LCOcoCo

L-  Il4  01  in C   Co   C -  01  COL C  O  C-  OLO  C OO  Co  Co m   "   1r

0 ~ CO   C O  C O>  t-   C OD 1  CO  01 CO   C-,   C o  - 0 1   Co C - C   01  C o  01  C O  Co Ol

N _   io   01  Co  0 4 1   0I   N   0  CoO _I  0 1 0 0 1 0 1 0 0 0 1

~  C o   o   C -  o   C O   1   C O   C o C O 0   C o - O  C O   m C o   C   C o -

_4t)NXNN N0ONOXOO       OONOCSaN

I   I  I  I  I  I  I  . C   .

ro4 N r

COO I?c:  j

CO
I IIIICOO  I CoI C

O:lCoCOCOOC XC

01O0COCOO      Co

""OWiLDOOO~ r-4 m

m - O C N 0  t- "
Ol-*CoC - OCO0  t'.oo

rt.lt.OCo CC

Co -4 LO

CO(oO

t- Ol r-
coO 01 c

1 111 1 11  1   ...- 1-

00CO COO
I  I  I  I  I I  .--  --

b 00

I I I I I I I    Cn0:

COC N

I   I   I   I   I   I   I  Cli (

N Co

N Mc     r_iN "   N O

CostCOoCoCo       C-C

("   O l C O Co0 0   1-   0 1 0 1

CoOC-COl-0        0101

C- C- Co

0100

Nl Nq N

CO  1 Co00

Co-4 C O

01 -                                  -I

C o0C O01 4cl -i  OC o  0 10Co

I I I I I I I  I a?  o o~

11111!   I  I. . .

C .                 .  .

. . . . .

~~~~~~~~~~~~~.     .   .   . .   .O .   . .   . .   .   . .   .   .   ..
t* *  W o  5  > ** ***  ; ****

0 ..

CC

C ;;4; S  ;x E ^h t 92t  A e m 3 E SF  m 5 i; t-   =3 PA  P., -

ClS0  0  * ** *  * *  *  * * * *  * *   p**+-

*       4+~~~~~~~~~~~~~~~4

5

czo

Ca,

Ca

00

0 ?

V C)

C_ Os

*3 .14-

TABLE 11I.-Incidence of Cancer of the Oesophagus and of the Cervix Uteri in

Selected Populations: Annual Rates per 100,000 Persons Aged 35-64 Years,
Standardized for Age.

Incidence of cancer of

Cervix
Oesophagus  uteri
Populations              (150)   (171)
AFRICA

S. Africa                        357 * 2

Transkei (African)

S. Rhodesia                      1575 5

Bulawayo (African)

AMERICA

New York City (Jewish)                     8- 8

,,  ,, ,, (Negro)               -       100- 6
,,  ,, ,, (Puerto Rican)                271-9
,,  ,, ,, (White non-Jewish)             38 5
ASIA

*Kazakhstan                      547-2 2

Ghurjev district

* Estimated from data for ages 30-59 years

Table III gives further data of particular interest for cancer of the oesophagus
in three populations (Kmet, personal communication; Rose, 1967; and Skinner,
1967) and for cancer of the cervix uteri in four populations (Haenszel and
Hillhouse, 1959).

Table IV gives incidence rates for primary cancer of the liver in forty-seven
populations over the age range 15 to 44 years. For this type of cancer, compari-
sons are made at younger ages, partly because liver cancer appears early in adult
life in areas where it is common and partly because there is less possibility of
confusion between primary and secondary cancers than at older ages when gastric,
colonic, bronchial, and gall bladder cancers are relatively frequent. Most of the
rates (33) have been reported specifically for primary cancer of the liver, but 14
have been estimated from the recorded mortality for cancer of the liver and biliary
passages or from incidence data that may include some other liver cancers. These
last have been included because examination of the rates in six populations
(Canada, Finland, Israel, New Zealand, Norway and Sweden) for which both
the mortality from cancer of the liver and biliary passages and the incidence of
primary liver cancer have been published, shows that, at ages 15 to 44 years, both
sets of rates are practically identical. It will be noted that all the estimated rates,
which may be regarded as maximal, are low.

All rates are shown for one sex only.* For comparison between countries this
is usually unimportant, as the sex ratio of most cancers is relatively constant from
one population to another. Cancer of the oesophagus-and to a less extent
cancer of the lung and cancer of the larynx-provide exceptions. For cancer of
the oesophagus the sex ratio at ages 35 to 64 years varies from less than 1-5 to 1
in England and Wales, Bombay, and Kazakhstan to 20 to 1 in France.

Further data that cannot be expressed in comparable arithmetrical form have
also been collected in an extensive review of the subject by Dunham and Bailar
(1968).

* Tables showing comparable rates for women can be obtained on application to Medical Research
Council's Statistical Research Unit.

6

R. DOLL

GEOGRAPHICAL DISTRIBUTION OF CANCER                            7

TABLE IV.-Incidence of Primary Liver Cancer in Different Populations:
Annual Rates Per 100,000 Men Aged 15-44 Years, Standardized for Age

Population         Incidence             Population         Incidence

AFRICA                                   EUROPE

Mozambique                               *Austria   .    .    .   .   0.1

Louren9o Marques  .    . 164-6         *Belgium   .    .    .   .   03
Nigeria                                  tDenmark   .    .    .   .   0 2

Ibadan   .    .   .    .   10 2         England and Wales

S. Africa                                  Birmingham region  .   .   01

Durban (African)  .    .   12-3          Liverpool region   .   .   03
Durban (Indian)   .    .   0- 7          S. Metropolitan region  .  0a2
Johannesburg (African)  .  10-2          S. Western region  .   .   0-0

*white .    .   .    .   0 6          Finland   .    .    .   .   0 3
Uganda                                   *Germany F.R.   .    .   .   0-2

Kyadondo      .   .    .   65          tIceland   .    .    .   .   0 3
AMERICA                                   *Ireland    .    .   .    .   01

Canada                                   *Italy .   .    .    .   .   04

Alberta  .    .            0 0          Netherlands

Manitoba          .        0 3            3 provinces  .    .   .   02
New Brunswick     .    .   0o0         Norway     .    .    .   .   0-1
Newfoundland .. O * O*Scotland                    .    .    .   .   04
Saskatchewan  .            0 .  0      Sweden     .    .    .   .   01
Chile . Sas1 1                           *Switzerland    .    .   .   04
Colombia                                 Yugoslavia Slovenia  .   .     1

Cali.    .   .    .    .   0-7        OCEANIA

Jamaica                                  *Australia  .   .    .   .   02

Kingston .    .   .    .   2-0          New Zealand    .    .   .   05
Puerto Rico    .    .    .   03          U.S.A., Hawaii

UT.S.A.                                    (Caucasian)   .    .   .   0.0

*non-white   .    .    .   10            (Hawaiian)    .    .   .   1 5
*white   .   .    .    .   0 2           (Japanese)    .    .   .   14
Connecticut  .    .    .   04
New York State    .    .   01
ASIA

India

tBombay .    .    .    .   0*1
Israel  .  .   .    .    .   0  5
*Japan      .   .    .    .   0-4
Singapore (Chinese)  .   .   41

* Estimated from the mortality rates for cancer of the liver and biliary passages (see text).

t Including, for Iceland, cancer of biliary passages and, for Denmark, cancer of the liver, primary
site unknown.

$ Estimated from the rate for ages 10-39 years by multiplying by 1-7.

I am grateful to Dr. P. Gregory for assistance in preparing the data for cancer
of the liver, and to Miss J. Allen and Miss B. Hafner for much of the calculation.

REFERENCES

BOMBAY CANCER REGISTRY.-(1966) 'Cancer in Greater Bombay, 1964'. The Bombay

Cancer Registry of the Indian Cancer Society, Bombay.

DOLL, R.-(1967) 'The prevention of cancer: pointers from epidemiology'. Nuffield

Provincial Hospitals Trust, London.-(1968) Tidsskr. norske Laegeforen. In press.
DOLL R. AND COOK, P.-(1967) Int. J. Cancer, 2, 269.

DUNHAM, L. J. AND BAILAR, J. C.-(1968) J. natn. Cancer Inst., 41, 155.

HAENSZEL, W. AND HTLLHOUSE, M.-(1959) J. natn. Cancer Inst., 22, 1157.

INTERNATIONAL UNION AGAINST CANCER.-(1966) 'Cancer incidence in five continents'.

Edited by R. Doll, P. Payne, and J. Waterhouse. U.I.C.C. Technical Report.
Berlin (Springer-Verlag).

8                                R. DOLL

MERKOVA, A. M., TSERKOVNOGO, G. F., AND KAUFMAN, B. D.-(1963) 'Morbidity and

mortality from malignant neoplasms in the U.S.S.R.' English edition edited by
J. G. Dean. London (Pitman Medical Publishing Co.).

ROSE, E. F.-(1967) Natn. Cancer Inst. Monogr. No. 25, p. 83.

SCHONLAND, M. AND BRADSHAW, E.-(1968) Int. J. Cancer, 3, 304.

SEGI, M.-(1960) 'Cancer mortality for selected sites in 24 countries (1950-1957)'.

Department of Public Health, Tohoku University School of Medicine, Sendai,
Japan.

SEGI, M. AND KURIHARA, M.-(1964) 'Cancer mortality for selected sites in 24 courLtries

No .3 (1960-1961)'. Department of Public Health, Tohoku University School of
Medicine, Sendai, Japan.

SKINNER, M. E. G.-(1967) Natn. Cancer Inst. Monogr. No. 25, p. 57.

WORLD HEALTH ORGANIZATION.-(1966) 'World Health Statistics Annual, 1963'.

Vol. 1. Geneva. (World Health Organization).

				


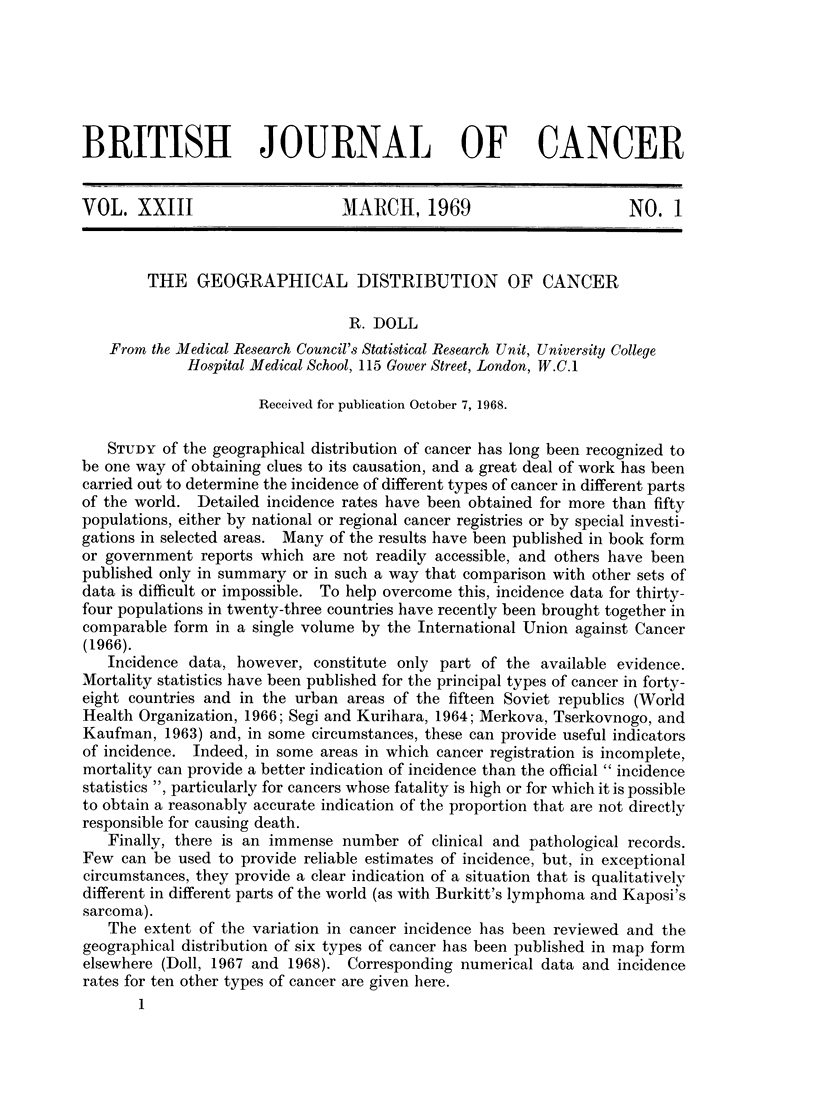

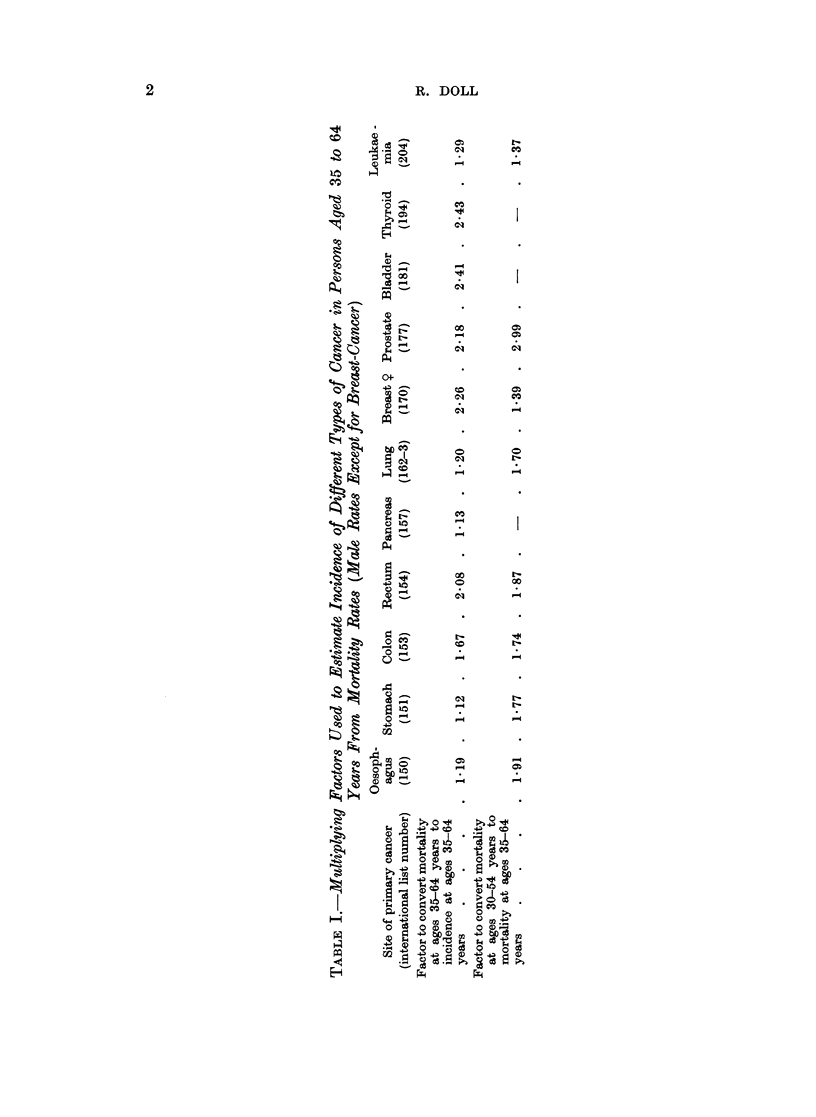

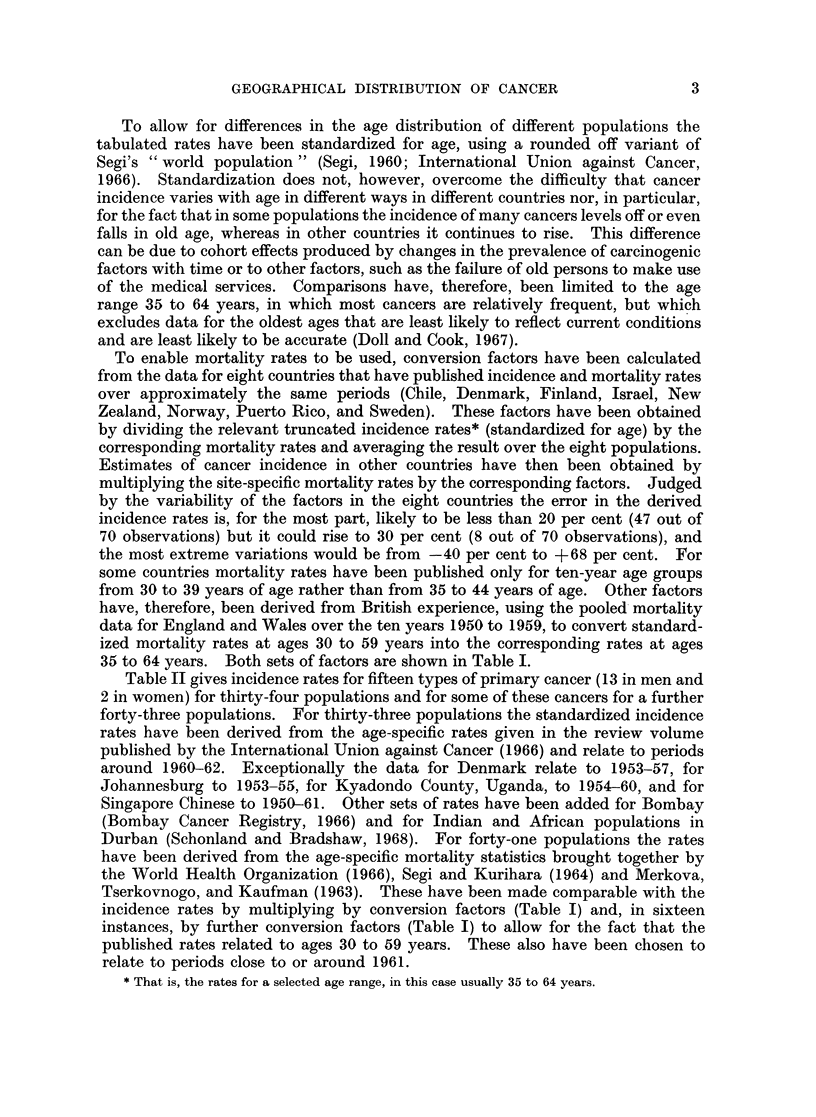

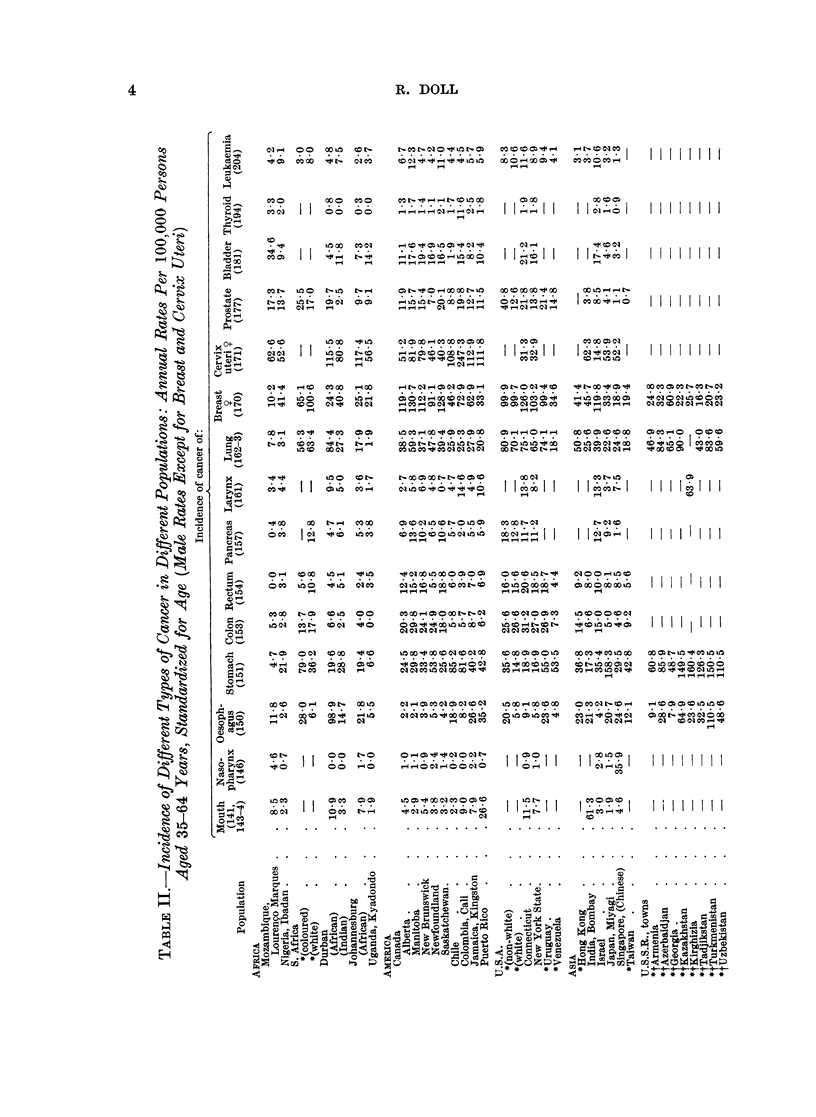

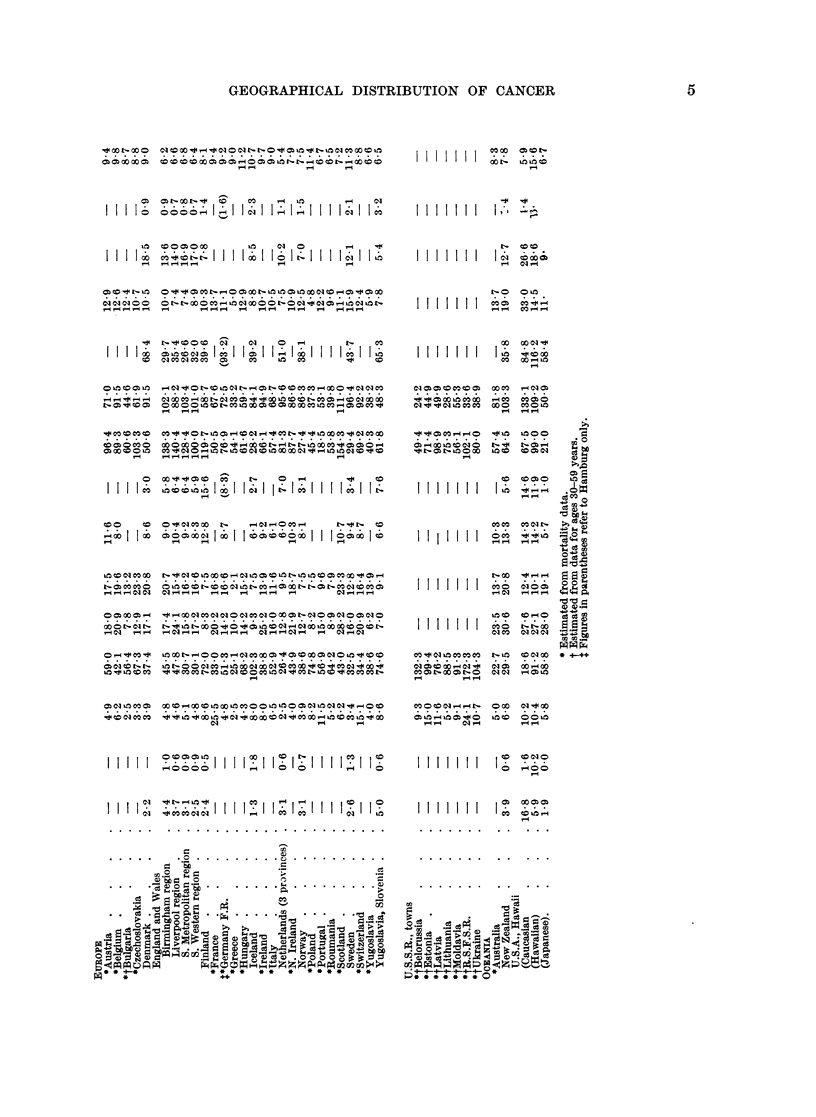

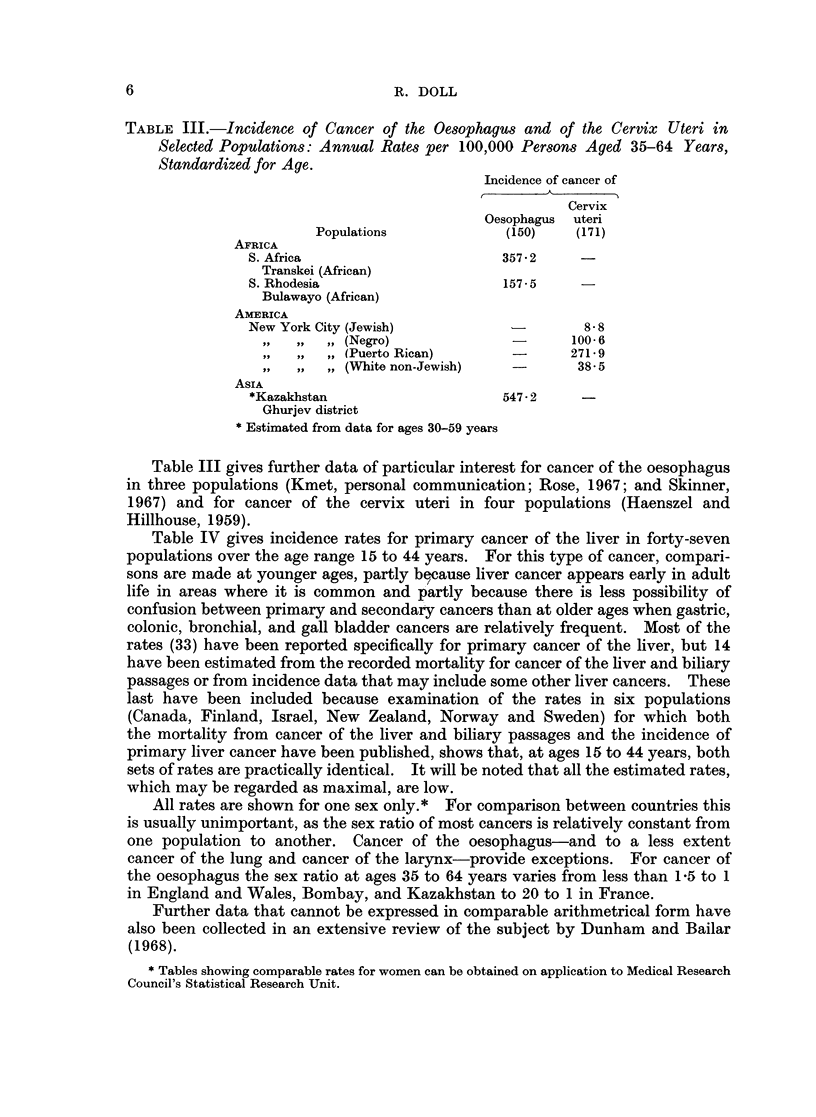

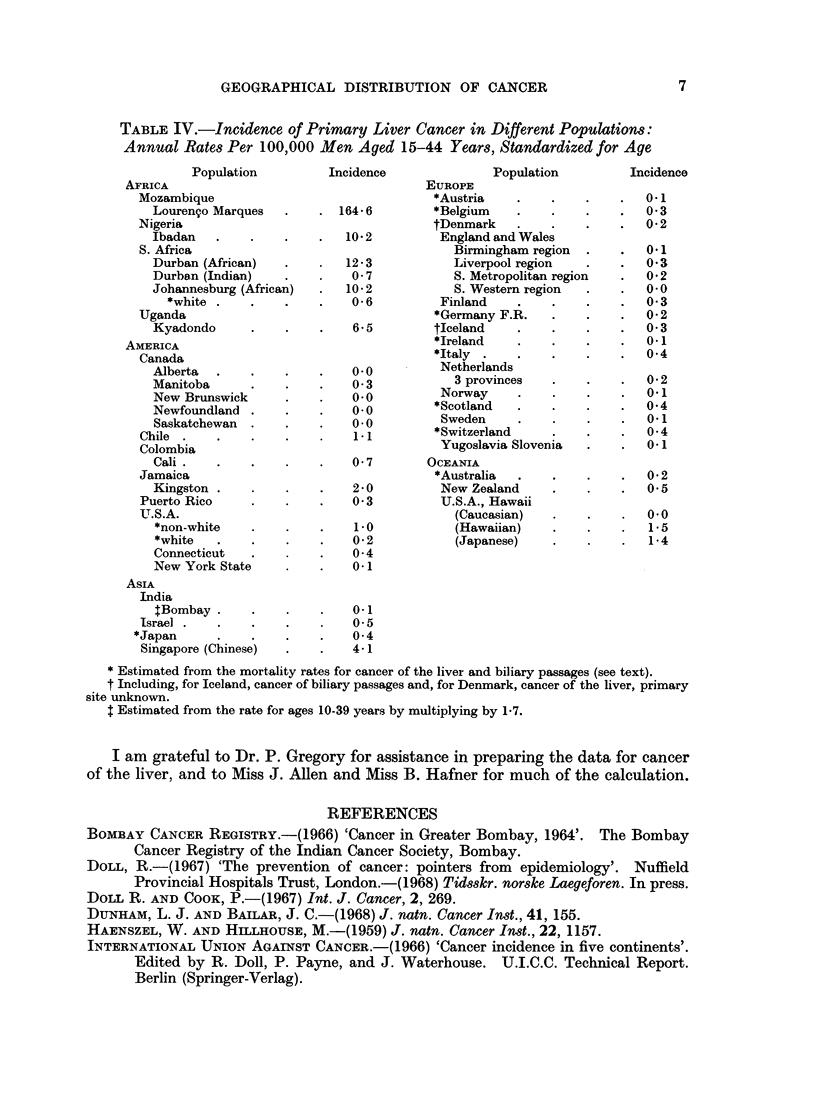

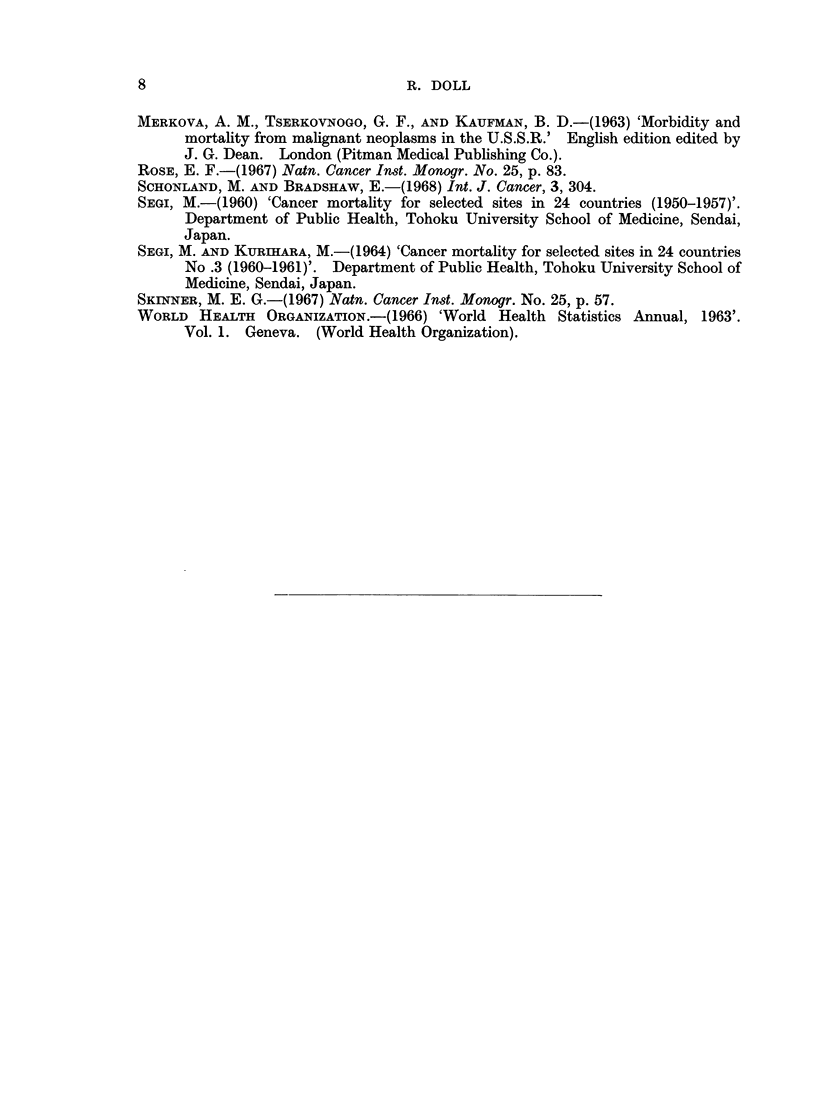

